# Effect of Moxibustion on the Serum Levels of MMP-1, MMP-3, and VEGF in Patients with Rheumatoid Arthritis

**DOI:** 10.1155/2020/7150605

**Published:** 2020-09-16

**Authors:** Zeyun Yu, Yingni Wang, Yuan Li, Chenxi Liao, Jingyang Dai, Yun Luo, Yuanzhang Hu, Siyu Tao, Jie Tang, Guanhua Chen, Ping Wu

**Affiliations:** ^1^Chengdu University of Traditional Chinese Medicine, Sichuan, Chengdu 610075, China; ^2^Hospital of Chengdu University of Traditional Chinese Medicine, Sichuan, Chengdu 610075, China; ^3^Chengdu Fifth People' Hospital, Chengdu 611130, China

## Abstract

**Background:**

Rheumatoid arthritis (RA) is a chronic inflammatory autoimmune disease, which will eventually lead to joints deformity and functional damage. The aim of this research is to evaluate the effect of moxibustion on the serum indicators related to bone and cartilage metabolism, matrix metalloproteinase 1 (MMP-1), matrix metalloproteinase 3 (MMP-3), and vascular endothelial growth factor (VEGF) in patients with RA and to explore the mechanism of moxibustion in the treatment of RA.

**Methods:**

We recruited 70 RA patients who met the inclusion criteria, and they were randomly divided into two groups, a treatment group and a control group in equal ratio. The control group took methotrexate, folate, or leflunomide orally, while the treatment group received methotrexate, folate, or leflunomide orally and moxibustion at ST36 (Zusanli), BL23 (Shen shu), and Ashi points. We compared the clinical symptoms, RA serological disease markers and serum contents of interleukin-1*β* (IL-1*β*), tumor necrosis factor-*α* (TNF-*α*), MMP-1, MMP-3, and VEGF of RA patients before and after treatment.

**Results:**

(1) The clinical symptoms and RA serological disease markers of the two groups improved after treatment (*P* < 0.05), while the clinical symptoms of the treatment group were significantly improved in comparison with the control group (*P* < 0.05). (2) The levels of IL-1*β*, TNF-*α,* and VEGF decreased in both groups after treatment (*P* < 0.05), but the treatment group was significantly decreased compared with the control group (*P* < 0.05). (3) There were significant differences in MMP-1 and MMP-3 contents after treatment in the treatment group (*P* < 0.05, *P* < 0.05), while there were no significant differences in the control group (*P* > 0.05, *P* > 0.05). Above all, the contents of IL-1*β*, TNF-*α*, MMP-1, MMP-3, and VEGF in the treatment group decreased more significantly than those in the control group (*P* < 0.05).

**Conclusion:**

The improvement effect of moxibustion on the clinical symptoms of RA patients may be related to influence on the contents of IL-1*β*, TNF-*α*, MMP-1, MMP-3, and VEGF, and moxibustion may play a potential role in bone protection.

## 1. Introduction

Rheumatoid arthritis (RA) is a chronic inflammatory autoimmune disease characterized by chronic synovial hyperplasia, which leads to progressive and irreversible destruction of articular cartilage and bone, finally resulting in joints deformity and loss of function [1]. Synovial tissue hyperplasia, inflammatory cell infiltration, pannus formation, and joints destruction are important pathological links in the pathogenesis of RA [2]. The incidence of RA in China is 0.19%–0.41%, and the prevalence is as high as 0.5%–1% all over the world [3]. Moreover, the incidence is increasing year by year. Despite RA bringing serious physical and mental damage and enormous economic burden to patients, it cannot be cured and most treatments only affect the progression or symptoms of RA [4].

VEGF is a high concentration of specific endothelial cell mitogen in RA synovial fluid and tissue, which is secreted by fibroblasts, keratinocytes, lymphocytes, and macrophages in RA synovium [5]. It can not only induce angiogenesis but also increase the local permeability of blood vessels, and promote the exudation of inflammatory factors, thus stimulating the formation of neovascularization, which will aggravate synovial inflammation and pannus formation, and eventually leads to joints swelling, pain, and deformation [6]. Fibroblast synovial cells (FLSs) also play a key role in the pathogenesis of rheumatoid arthritis by producing inflammatory cytokines and proteases [7], such as matrix metalloproteinases (MMPs), and can cause permanent damage to joints. It has been found that MSC-derived miR-150-5p exosome (Exo-150) decreased migration and invasion in RA-FLS and downregulated tube formation in human umbilical vein endothelial cells (HUVECs) by targeting MMP14 and VEGF. Injection of Exo-150 reduced the thickness of the hindfoot and clinical arthritis score in collagen-induced arthritis mice [8]. In addition, MMP-1, MMP-2, MMP-9, and MMP-13 are closely related to the expression of RA-FLS and VEGF [9, 10], but there are relatively few studies on the correlation between MMP-1, MMP-3, and VEGF, while MMP-1 and MMP-3 are closely related to the bone destruction of RA joints [11].

Moxibustion is a kind of physiotherapy, which has good anti-inflammatory, analgesic, and immunomodulatory effects. Relevant studies have shown that moxibustion can not only repair the central mechanism of RA inflammatory injury, reduce the release of local inflammatory mediators [12], but also regulate the expression of peripheral cytokines and the signal transduction pathway of peripheral inflammatory cells, such as hypoxia-inducible factor-1*α* (HIF-1*α*) and toll-like receptor 4/nuclear factor-*κ*B [13–15]. Moxibustion has no side effects compared with taking conventional medicine. Therefore, in this study, we from the perspective that “moxibustion regulates VEGF and inhibits the formation of synovial pannus by affecting the serum levels of MMP-1 and MMP-3, thus alleviating synovial inflammation and articular cartilage destruction in RA patients” observe the effect of moxibustion on the contents of MMP-1, MMP-3, and VEGF and explore the response of moxibustion to synovial inflammation as well as its potential bone protective effect in the treatment of RA.

## 2. Patients and Methods

### 2.1. Sample Collection

**Figure 1:**
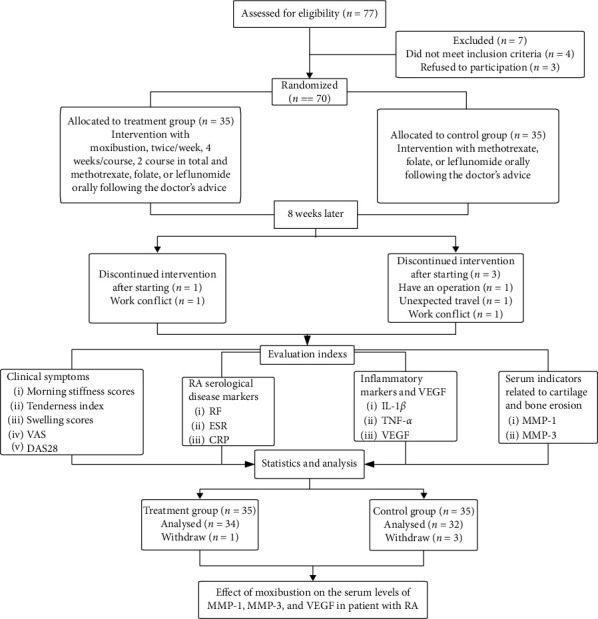
Consort diagram of the study.

We recruited 70 RA patients from March 2017 to October 2018 in the Hospital of Chengdu University of Traditional Chinese Medicine to receive treatment. 70 RA patients were recruited in accordance with the following inclusion criteria, they were randomly assigned to a treatment group or a control group in equal ratio (Figure 1). The research was in compliance with the Declaration of Helsinki and was approved by the Sichuan Regional Ethics Review Committee of Traditional Chinese Medicine (No. 2015KL-05). The information of this study was explained to all recruited participants, and written informed consent was obtained from each participant.

#### 2.1.1. Inclusion Criteria

The patients participated in this study should meet all the following conditions:

1. Diagnosed with RA, RA was defined as fulfilling the 2010 American College of Rheumatology (ACR) and European League against Rheumatism (EULAR) [16, 17]
2. Age from 18 to 65 years old
3. DAS28 (disease activity score of 28 joints) > 3.2
4. With clear consciousness and able to cooperate with this study
5. Receive no other antirheumatic medication within the previous 6 months
6. Voluntarily participate in this study and sign the informed consent

#### 2.1.2. Exclusion Criteria

The patients will be excluded from this research if they belong to one of the following conditions:

1. Advanced patients with severe deformity of joints, and the function is in stage IV
2. Overlap with other autoimmune diseases, such as systemic lupus erythematosus and Sjogren's syndrome.
3. Combine with malignant tumors or other serious diseases
4. Women going through pregnancy or lactation
5. Being afraid of the moxibustion treatment
6. Allergic to moxa-smog and a variety of drugs

### 2.2. Randomization and Blinding

Participants were randomly assigned to a control group or a treatment group using a computerized random number generated by SPSS21.0 software (SPSS, Inc., Chicago, Illinois, USA). The allocation concealment was enclosed in sealed, opaque, sequentially numbered envelopes; the envelopes were numbered as 1–70 integers, and then the screened RA patients were assigned envelopes with corresponding serial numbers in the order of treatment. The purpose of this research is to study the mechanism of the synergistic effect of moxibustion on conventional medicine, as it is easy to know whether the patient has received moxibustion treatment or not after we collected serum indicators twice; it is impossible to blind the patients and the moxibustion doctors. In order to eliminate potential bias, we have blinded the recruitment doctor, data collectors, and data statisticians.

### 2.3. Interventions

**Figure 2:**
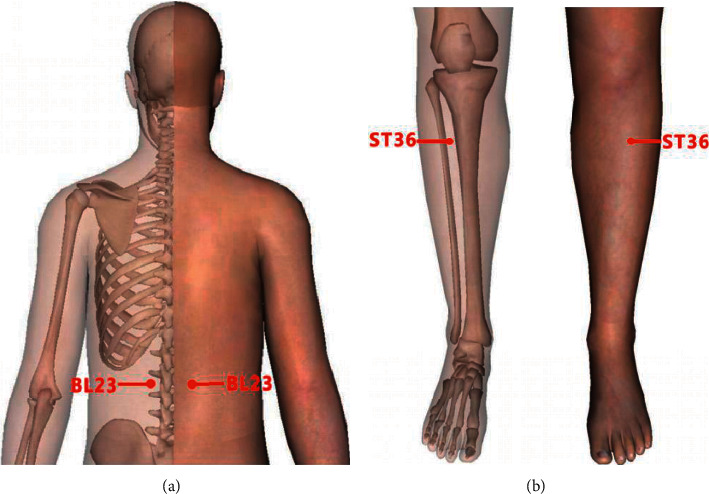
Acupoints: (a) BL23 (Shen shu) point is located under the spinous process of the second lumbar vertebra, 1.5 inches beside the posterior midline. (b) ST36 (Zusanli) point is 3 inches under the eye of the outer knee and about 1 inch from the anterior edge of the tibia, and (Ashi) points are located where swelling and paining occur.

All patients included in this study were treated with conventional medicines following the doctor's advice and long-term oral therapy. Their treatment plan was taking methotrexate (7.5 mg, once a week), folate (10 mg, once a week), or leflunomide (10 mg, once a day) orally, while the patients in the treatment group were treated with moxibustion at ST36 (Zusanli), BL23 (Shen shu), and Ashi points on this basis. The points were based on the National Standard of the People's Republic of China (GB/T12346- 2006). The name and positioning of acupoints are shown in Figure 2. “BL23 (Shen shu)” was treated with indirect moxibustion, moxibustion 3-4 cones, with the patient's skin flush as the degree. “ST36 (Zusanli)” and “Ashi” points were treated with direct moxibustion for 5–7 cones every time. The treatment group patients received moxibustion therapy twice a week, 4 weeks as a course of treatment, with a total of 2 courses. Moxibustion was performed by licensed-TCM doctors with over 3 years' experience of clinical practice.

**Figure 3:**
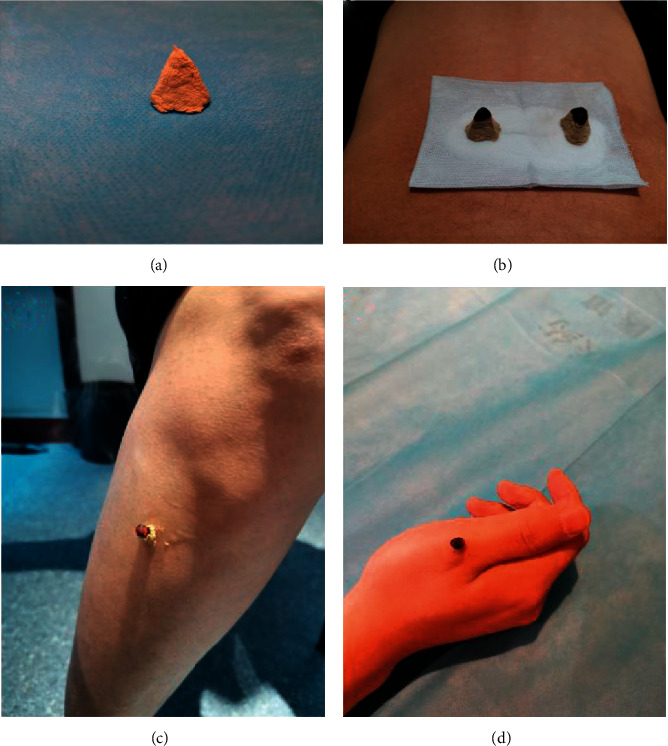
Diagram of moxibustion. (a) The shape of moxa. (b) The patient was treated at the BL23 (Shen shu) point. (c) The patient was treated at the ST36 (Zusanli) point. (d) The patient was treated at the (Ashi) point.

Doctors labeled the acupoints with a marker, and they will put a piece of sterile gauze on the BL23 (Shen shu) points, and then the right amount of salts will be put on the gauze (Figure 3(b)). Besides, the doctor will smear a little Vaseline at ST36 (Zusanli) and Ashi acupoints to stick moxa. The moxa of indirect moxibustion was made with a mold using mugwort floss, and its shape is a cone about 1 cm in diameter and 1 cm in height (Figure 3(a)). The moxa of direct moxibustion was handmade using mugwort floss, and it is about the same size and shape as half a jujube pit. Moxa was affixed to the acupoint and then it ignites the top of the moxa cone (Figures 3(c) and 3(d)). The cone was lifted up quickly and put another one if the patient felt causalgia during the treatment.

### 2.4. Evaluation Methods

We will evaluate the following indicators before and after treatment.

#### 2.4.1. Clinical Symptoms

Joints tenderness index, joints swelling index, joints morning stiffness score, visual analogue scale [18] (VAS: a standard tool for measuring pain intensity in chronic pain studies. 0, no pain; 10, severe pain), and disease activity score of 28 joints [19] (DAS28: assessment of RA disease activity by using 28 tender and swollen joint count disease activity score and ESR).

#### 2.4.2. RA Serological Disease Markers

Some serological markers are as follows: rheumatoid factor (RF), erythrocyte sedimentation rate (ESR), and C-reactive protein (CRP).

####  2.4.3. Related Inflammatory Factors and Bone Metabolic Indexes

The levels of IL-1*β*, TNF-*α*, MMP-1, MMP-3, and VEGF in serum.

### 2.5. Specimen Collection

We extracted 3–5 ml of the patient's elbow venous blood before and after treatment. Blood was allowed to clot at room temperature for 15 min, and serum was obtained by centrifugation at 3000 ×*g* for 5 min and then immediately stored at −80°C for reserve. After patients completed treatment, the serum samples were sent to Chengdu Lilai Biomedical Experimental Center for testing, and enzyme-linked immunosorbent assay (ELISA) was used to detect the contents of MMP-1, MMP-3, and VEGF.

### 2.6. Statistical Analysis

Data were analyzed using the SPSS version 21.0 software. The count data were tested by the chi-square (*χ*^2^) test, and the measurement data were expressed as mean ± standard deviation (*x* ± *s*). The normal distribution was satisfied by the *t* test, data in each group were analyzed by using the paired-samples *t* test, while the analysis was performed between the two groups using independent samples *t* test. Nonparametric test was used for nonnormal distribution of measurement data, and data in each group were analyzed by using Wilcoxon signed-rank sum test, while the analysis was performed between the two groups using the Mann–Whitney *U* test. The value of *P* < 0.05 was considered to be statistically significant.

## 3. Results

### 3.1. Baseline Characteristics

A total of 66 patients completed this study. The control group consisted of 32 subjects, 4 men, and 28 women (with an average age of 48.19 ± 9.87 years, average course of disease was 6.23 ± 6.13 years). There were 34 patients in the treatment group, including 3 men and 31 women (with an average age of 47.29 ± 10.47 years, average course of disease was 7.63 ± 7.24 years). There was no significant difference in general information between the two groups (*P* > 0.5).

The baseline characteristics of clinical symptoms and RA serological disease markers of both the control group and treatment group are shown in Table 1.

After treatment, the clinical symptoms and RA serological disease markers of both the control group and treatment group changed, as it is shown in Table 2.

### 3.2. The Clinical Symptoms and RA Serological Disease Markers

After treatment, the tenderness index, swelling index, morning stiffness score, VAS, and DAS28 of the two groups were significantly improved (*P* < 0.01), indicating that clinical symptoms were improved after treatment. However, after 8 weeks of treatment, clinical symptoms of the treatment group were significantly improved compared to the control group (*P* < 0.05) (Table 2).

There was significant improvement in ESR, CRP, and RF in both groups after treatment (CRP and RF, treatment group, *P* < 0.01; control group, *P* < 0.05; ESR, both groups *P* < 0.01), which indicated that ESR, CRP, and RF of RA patients in both groups decreased after treatment. However, intergroup comparisons did not show significant difference in ESR, CRP, and RF contents after treatment (*P* > 0.05), indicating that there was no significant difference in the improvement of RA serological disease markers between the two groups of RA patients (Table 2).

The changes of contents of IL-1*β*, TNF-*α*, MMP-1, MMP-3, and VEGF of both control group and treatment group before and after treatment are shown in Table 3.

### 3.3. The Contents of IL-1*β*, TNF-*α,* and VEGF

The contents of IL-1*β* and VEGF decreased in both groups after treatment (treatment group, *P* < 0.01; control group, *P* < 0.05), and the levels of TNF-*α* decreased in both groups after treatment (*P* < 0.05). While compared to the control group, the contents of IL-1*β*, TNF-*α,* and VEGF in the treatment group were significantly lower than those in the control group; intergroup comparisons show significant differences (*P* < 0.05) (Table 3).

### 3.4. The Contents of MMP-1 and MMP-3

There were significant differences in MMP-1 and MMP-3 contents before and after treatment in the treatment group (*P* < 0.05, *P* < 0.05), while there were no significant differences in the control group (*P* > 0.05, *P* > 0.05). After 8 weeks of treatment, the changes of MMP-1 and MMP-3 contents in the treatment group were significantly better than those in the control group (*P* < 0.05) (Table 3).

## 4. Discussion

Rheumatoid arthritis (RA) is a disease with high disability and high fatality rate, and there is no safe and effective treatment in modern medicine. Conventional drugs treatment can lead to liver function damage, kidney function damage, osteoporosis, etc. [20]. At present, moxibustion is gradually beginning to be used in the treatment of RA, and it can regulate the immune and inflammatory response of the body by regulating the hypothalamus-pituitary-adrenal cortex axis [12]. However, the mechanism of moxibustion in treating RA is very complex, involving cytokines, signal pathways, protein expression, neuroregulation, and so on [15]. Most of the studies on the efficacy and mechanism of moxibustion are laboratory studies, and there are relatively few studies on clinical mechanism. Therefore, we have begun to study the clinical mechanism in recent years [21, 22]; this research chooses MMP-1, MMP-3, and VEGF as the research objects, which is helpful to further reveal the effective mechanism of moxibustion in the treatment of RA.

### 4.1. The Effect of Moxibustion on Clinical Symptoms and RA Serological Disease Markers of Patients with RA

The results of this study showed that the clinical symptoms of both groups were improved after treatment, but moxibustion combined with conventional medicine improved more obvious. However, there was no significant difference in the improvement of RA serological disease markers between the two groups after treatment; we speculated that it may be related to the complexity of RA as a chronic autoimmune disease. In general, the improvement of laboratory indexes of chronic diseases is not obvious in a short period of treatment. From the clinical symptoms of RA patients, aspects of improvement were obvious but changes in RA serological disease markers may take longer time to observe.

ST36 and BL23 are commonly used for acupuncture and moxibustion in the treatment of RA. Our previous basic researches and clinical studies have shown that moxibustion at ST36, BL23, and Ashi points can significantly improve the clinical symptoms of RA patients, and its mechanism may be related to the regulation of cytokines and signal pathways, such as IL-1*β*, TNF-*α*, HIF-1*α*, and NF- kB [22, 23]. Besides, some studies have confirmed that moxibustion at ST36 and BL23 could improve synovitis by regulating the RANKL/OPG signaling pathway and IL-17 [24, 25]. So, the improvement of clinical symptoms of RA patients by moxibustion may be related to the anti-inflammatory and analgesic effects of moxibustion and the regulation of autoimmunity by acupoints, and moxibustion at ST36, BL23, and Ashi points can regulate a variety of cytokines and signal pathways; its internal mechanism is worthy of further discussion.

### 4.2. The Effect of Moxibustion on IL-1*β*, TNF-*α*, MMP-1, MMP-3, and VEGF in Serum of RA Patients

The results of our study showed that there was significant decrease in IL-1*β*, TNF-*α,* and VEGF contents in both groups after treatment, but the decrease in the treatment group was more obvious than those in the control group. The contents of MMP-1 and MMP-3 in the treatment group were significantly different after treatment, but there were no significant differences in the control group. According to the TCM theory, the mechanism may be related to the effect of moxibustion on warming meridians and dispelling cold, promoting the blood circulation and removing the blood stasis. ST36 can regulate its own disordered immune system by stimulating vital qi; BL23 can tonify the kidney essence and the kidney-yang, thus nourishing bone marrow and promoting the restoration of joint movement.

In the pathogenesis of RA, the most prominent manifestation of RA is synovial inflammation and pannus formation. IL-1*β* has a strong proinflammatory effect; it can promote the development of synovial inflammation and induce bone resorption [26, 27]. TNF-*α* is closely related to the occurrence and development of synovitis in RA; it is the main proinflammatory cytokine and plays an important role in local synovial inflammation, pannus formation, and tissue injury in RA patients [28], and both of TNF-*α* and IL-1*β* can stimulate VEGF expression [29, 30]. VEGF can induce vascular proliferation and further stimulate the formation of neovascularization, thus aggravating synovial inflammation and pannus formation [6]. MMPs can not only reduce almost all extracellular matrix components except polysaccharide, degrade collagen, proteoglycan, and other extracellular matrixes in articular cartilage and bone RA, but also promote the erosion of pannus on articular cartilage. It may play a key role in the destruction of articular cartilage in RA [31].

The concentration of VEGF in synovial fluid and synovial tissue of patients with RA was increased, and the levels of VEGF was positively correlated with the development of pannus. MMP-1 is significantly upregulated in the cartilage of RA and possibly play an important role in the degradation of collagen in the tissue of RA [32]. MMP-3 as a specific marker of synovitis in RA, we can detect the increase of MMP-3 levels in serum no matter in the early stage of RA disease or in the late stage of bone destruction [33]. It can significantly upregulate the expression of other MMPs and activate other collagenase, promoting the formation of pannus and the destruction of cartilage [34, 35].

Modern medicine can temporarily delay the development of RA, but the adverse effects are obvious. Moxibustion is a traditional Chinese therapy that relies on the heat from burning moxa to be transferred beneath the skin surface [36]. At present, the clinical research of moxibustion in the treatment of RA mainly focused on anti-inflammation and analgesia, but the key pathological changes of synovial pannus formation and cartilage damage have not been studied more deeply and comprehensively. The results of our study showed that moxibustion could reduce the contents of MMP-1 and MMP-3 and regulate VEGF to inhibit the formation of synovial pannus in patients with RA, thus alleviating synovial inflammatory reaction and articular cartilage destruction in RA patients. It may have a potential bone protective effect.

## 5. Conclusions

Moxibustion at ST36, BL23, and Ashi points can improve the clinical symptoms of RA patients. Its internal mechanism and target may be related to the fact that moxibustion can reduce the contents of IL-1*β*, TNF-*α*, MMP-1, MMP-3, and VEGF in serum of RA patients. Meanwhile, moxibustion may improve the structure of synovium in patients with RA.

Although MMP-1 and MMP-3, the key factors of articular cartilage, and bone destruction have been studied here, whether the treatment of RA by moxibustion can reverse articular cartilage and bone destruction have not been confirmed by relevant imaging examinations in this study. Thus, further study using imaging data to confirm whether moxibustion can reverse or delay the destruction of articular cartilage and bone in RA patients is required.

## Figures and Tables

**Table 1:** Baseline characteristics.

Outcome measure	Treatment group (*n* = 34)	Control group (*n* = 32)	*P* value
Clinical symptoms			
Morning stiffness score	3.29 (1.96)	3.25 (1.67)	0.99^†^
Tenderness index	13.47 (7.80)	13.75 (6.97)	0.88^†^
Swelling index	8.97 (6.13)	8.78 (6.26)	0.90^†^
VAS	6.56 (1.46)	6.69 (1.66)	0.74^†^
DAS28	5.99 (1.20)	5.98 (1.01)	0.96^†^

RA serological disease markers			
ESR (mm/60 min)	52.85 (30.20)	53.66 (31.37)	0.92^Δ^
CRP (mg/L)	16.80 (29.70)	20.56 (29.27)	0.56^Δ^
RF (IU/ml)	167.09 (197.67)	191.58 (215.65)	0.41^Δ^

Values are represented as mean (SD). ^†^*P* value by independent samples *t* test. ^Δ^*P* value by Mann–Whitney *U* test. Table 1 shows there were no significant differences in baseline characteristics between the two groups (*P* > 0.05).

**Table 2:** Clinical symptoms and RA serological disease markers after treatment.

Outcome measure	Treatment group (*n* = 34)	Control group (*n* = 32)	*P* value
Clinical symptoms			
Morning stiffness score	1.32 (1.27)	2.44 (1.66)	0.005^†^
Tenderness index	6.15 (5.00)	10.25 (7.22)	0.011^†^
Swelling index	3.09 (3.04)	6.53 (6.49)	0.042^†^
VAS	3.32 (1.65)	4.72 (1.92)	0.002^†^
DAS28	4.40 (1.26)	5.19 (1.39)	0.017^†^

RA serological disease markers			
ESR (mm/60 min)	38.47 (26.92)	41.88 (27.36)	0.612^Δ^
CRP (mg/L)	7.90 (8.40)	7.06 (7.31)	0.668^Δ^
RF (IU/ml)	130.22 (152.09)	158.58 (195.78)	0.549^Δ^

Values are represented as mean (SD). ^†^*P* value by independent samples *t* test. ^Δ^*P* value by Mann–Whitney *U* test.

**Table 3:** Changes of contents of IL-1*β*, TNF-*α*, MMP-1, MMP-3, and VEGF.

Outcome measures	Treatment group (*n* = 34)	Control group (*n* = 32)	*P* value
IL-1*β* (pg/mL)			
Baseline	30.75 (16.31)	33.52 (16.34)	0.493^†^
Posttherapy	21.75 (10.61)	28.56 (15.69)	0.042^†^

TNF-*α* (pg/mL)			
Baseline	27.66 (14.12)	20.60 (12.09)	0.204^†^
Posttherapy	32.43 (16.01)	27.07 (13.41)	0.043^†^

MMP-1 (pg/mL)			
Baseline	2.85 (1.68)	3.69 (2.11)	0.076^†^
Posttherapy	2.38 (1.36)	3.39 (1.72)	0.010^†^

MMP-3 (pg/mL)			
Baseline	141.40 (77.16)	160.11 (61.98)	0.283^†^
Posttherapy	120.77 (64.52)	152.18 (58.00)	0.042^†^

VEGF (pg/mL)			
Baseline	87.43 (47.65)	109.84 (55.74)	0.083^†^
Posttherapy	66.27 (33.46)	91.18 (47.49)	0.016^†^

Values are represented as mean (SD). ^†^*P* value by independent samples *t* test.

## Data Availability

The data used to support the findings of this study are available from the corresponding author upon reasonable request.

## References

[1] Bai J., Ge G., Wang Y. (2019). A selective CB2 agonist protects against the inflammatory response and joint destruction in collagen-induced arthritis mice. *Biomedicine & Pharmacotherapy*.

[2] Tong-Lie H., Nan M., Jin-Tao G. (2019). DDR2-CYR61-MMP1 signaling pathway promotes bone erosion in rheumatoid arthritis through regulating migration and invasion of fibroblast-like synoviocytes. *Journal of Bone and Mineral Research*.

[3] Wang G., Xu H., Mu R. (2015). Management of rheumatoid arthritis in people’s republic of China &ndash; focus on tocilizumab and patient considerations. *International Journal of General Medicine*.

[4] Uhlig T., Moe R. H., Kvien T. K. (2014). The burden of disease in rheumatoid arthritis. *PharmacoEconomics*.

[5] Hae-Rim K., Kim K.-W., Kim B.-M. (2015). The effect of vascular endothelial growth factor on osteoclastogenesis in rheumatoid arthritis. *PLoS One*.

[6] Ikeda M., Hosoda Y., Hirose S. (2015). Expression of vascular endothelial growth factor isoforms and their receptors Flt-1, KDR, and neuropilin-1 in synovial tissues of rheumatoid arthritis. *Journal of Pathology*.

[7] Xu Q., Yin S., Yao Y. (2019). MAST3 modulates the inflammatory response and proliferation of fibroblast-like synoviocytes in rheumatoid arthritis. *International Immunopharmacology*.

[8] Chen Z., Wang H., Xia Y., Yan F., Lu Y. (2018). Therapeutic potential of mesenchymal cell-derived miRNA-150-5p-expressing exosomes in rheumatoid arthritis mediated by the modulation of MMP14 and VEGF. *The Journal of Immunology*.

[9] Yuan H., Yang P., Zhou D. (2014). Knockdown of sphingosine kinase 1 inhibits the migration and invasion of human rheumatoid arthritis fibroblast-like synoviocytes by down-regulating the PI3K/AKT activation and MMP-2/9 production in vitro. *Molecular Biology Reports*.

[10] Kim K. S., Choi H. M., Lee Y.-A. (2011). Expression levels and association of gelatinases MMP-2 and MMP-9 and collagenases MMP-1 and MMP-13 with VEGF in synovial fluid of patients with arthritis. *Rheumatology International*.

[11] Inoue K., Masuko-Hongo K., Okamoto M., Nishioka K. (2005). Induction of vascular endothelial growth factor and matrix metalloproteinase-3 (stromelysin) by interleukin-1 in human articular chondrocytes and synoviocytes. *Rheumatology International*.

[12] Tian-Tian Z., Zhong-Ting Z., Yi-Kun Z. (2017). Review on modern repair mechanism of moxibustion for treating inflammatory damage of rheumatoid arthritis. *Zhen Ci Yan Jiu*.

[13] Zheng B., Hu L., Song X. (2014). Analgesic effect of different moxibustion durations in rheumatoid arthritis rats. *Journal of Traditional Chinese Medicine*.

[14] Zhang C.-Y., Hu L., Cai R.-L. (2018). Toll-like receptor 4/nuclear factor-*κ*b signaling in synovial tissue is involved in the anti-inflammatory effect of moxibustion in rats with rheumatoid arthritis. *Zhen Ci Yan Jiu*.

[15] Hu W.-B., Luo L., Hu L. (2012). Study on the effect of moxibustion in treating rhreumatoid arthritis rats and its mechanism. *Journal of Acupuncture and Tuina Science*.

[16] Funovits J., Aletaha D., Bykerk V. (2010). The 2010 American college of rheumatology/european league against rheumatism classification criteria for rheumatoid arthritis: phase 2 methodological report. *Arthritis & Rheumatism*.

[17] Daniel A., Tuhina N., Silman Alan J. (2010). Rheumatoid arthritis classification criteria: an American college of rheumatology/european league against rheumatism collaborative initiative. *Annals of the Rheumatic Diseases*.

[18] Jensen M., Chen C., Brugger Andrew M. (2003). Interpretation of visual analog scale ratings and change scores: a reanalysis of two clinical trials of postoperative pain. *The Journal of Pain*.

[19] Fransen J., Creemers M. C. W., Van Riel P. L. C. M. (2004). Remission in rheumatoid arthritis: agreement of the disease activity score (DAS28) with the ARA preliminary remission criteria. *Rheumatology*.

[20] Ain Q., Zeeshan M., Khan S., Ali H. (2019). Biomimetic hydroxyapatite as potential polymeric nanocarrier for the treatment of rheumatoid arthritis. *Journal of Biomedical Materials Research Part A*.

[21] Yan X., Yu B., Yuan L. I. (2019). Effect of moxibustion on VEGF and IL-1*β* in patients with rheumatoid arthritis. *Chinese Archives of Traditional Chinese Medicine*.

[22] Gong Y., Yu Z., Wang Y. (2019). Effect of moxibustion on HIF-1*α* and vegf levels in patients with rheumatoid arthritis. *Pain Research and Management*.

[23] Tang Y., Yu B., Wang Y. (2019). Study on the effect of moxibustion on NIK/NF-*κ* B/VEGF pathway and the mechanism of anti-inflammatory and analgesic effect in patients with RA. *Lishizhen Medicine and Materia Medica Research*.

[24] Chen Y., Li H., Luo X. (2019). Moxibustion of zusanli (ST36) and shenshu (BL23) alleviates cartilage degradation through RANKL/OPG signaling in a rabbit model of rheumatoid arthritis. *Evidence-Based Complementary and Alternative Medicine*.

[25] Zhao C., Li X., Yang Y. (2019). An analysis of treg/Th17 cells imbalance associated microRNA networks regulated by moxibustion therapy on zusanli (ST36) and shenshu (BL23) in mice with collagen induced arthritis. *American Journal of Translational Research*.

[26] Kato T., Miyaki S., Ishitobi H. (2014). Exosomes from IL-1*β* stimulated synovial fibroblasts induce osteoarthritic changes in articular chondrocytes. *Arthritis Research & Therapy*.

[27] Zhang B., Jiang W. (2019). IL-1*β*, IL-17A, CRP and biologics history might serve as potential markers for clinical response to etanercept in rheumatoid arthritis patients. *Inflammopharmacology*.

[28] Moelants E. A., Mortier A., Van Damme J., Proost P. (2013). Regulation of TNF-*α* with a focus on rheumatoid arthritis. *Immunology & Cell Biology*.

[29] Tipton D. A., Christian J., Blumer A. (2016). Effects of cranberry components on IL-1*β*-stimulated production of IL-6, IL-8 and vegf by human tmj synovial fibroblasts. *Archives of Oral Biology*.

[30] Tao L., Xue J. F. (2018). Effects of TNF-*α* in rheumatoid arthritis via attenuating *α*1 (i) collagen promoter. *European Review for Medical and Pharmacological Sciences*.

[31] Sabeh F., Fox D., Weiss S. J. (2010). Membrane-type I matrix metalloproteinase-dependent regulation of rheumatoid arthritis synoviocyte function. *The Journal of Immunology*.

[32] Wu H., Du J., Zheng Q. (2008). Expression of MMP-1 in cartilage and synovium of experimentally induced rabbit ACLT traumatic osteoarthritis: immunohistochemical study. *Rheumatology International*.

[33] Van Hove I., Lemmens K., Van de Velde S., Verslegers M., Moons L. (2012). Matrix metalloproteinase-3 in the central nervous system: a look on the bright side. *Journal of Neurochemistry*.

[34] Huang J., Xie B., Li Q. (2013). Infliximab reduces CD147, MMP-3, and MMP-9 expression in peripheral blood monocytes in patients with active rheumatoid arthritis. *European Journal of Pharmacology*.

[35] Houseman M., Potter C., Marshall N. (2012). Baseline serum MMP-3 levels in patients with rheumatoid arthritis are still independently predictive of radiographic progression in a longitudinal observational cohort at 8 years follow up. *Arthritis Research & Therapy*.

[36] Maxim S., Deng H.-A., Sheu Tony W. H. (2020). Experimental and numerical study on the temperature elevation in tissue during moxibustion therapy. *Evidence-Based Complementary and Alternative Medicine*.

